# Stopping the Vicious Cycle: Equitable Enforcement Strategies to Achieve Safe, Stable, and Accessible Housing for People with Disabilities

**DOI:** 10.1111/1468-0009.12683

**Published:** 2024-01-14

**Authors:** KATIE HANNON MICHEL, MAYA HAZARIKA WATTS, JESSICA BRESLIN, ELIZABETH TOBIN‐TYLER

**Affiliations:** ^1^ ChangeLab Solutions; ^2^ School of Public Health Brown University

**Keywords:** disability, health law, housing

## Abstract

**Policy Points**

- People with disabilities experience a vicious cycle of poverty, poor health, and marginalization partly because of the inequitable implementation and enforcement of laws, including *underenforcement* of civil rights and housing laws and *overenforcement* of punitive nuisance and criminal laws.
- Inequitable enforcement reflects policy choices that prioritize powerful entities (e.g., landlords, developers) to the detriment of people who experience intersectional structural discrimination based on, for example, race, disability, and income.
- Equitable enforcement, a process of ensuring compliance with the law while considering and minimizing harms to marginalized people, can promote health and disability justice by increasing access to safe, stable, and accessible housing.

The law is widely recognized as a structural driver of health.^1^ The law affects health not only through its substance and design but also through how it is implemented and enforced by law enforcement and other government officials.^2^ People with both physical and mental health disabilities suffer negative health outcomes through *underenforcement*, in which they are disproportionately underserved by laws designed to promote their health, safety, and access to opportunity, and through *overenforcement*, in which they are disproportionately affected by punitive enforcement approaches.

Lack of access to safe, stable, and affordable housing is reaching a crisis point in communities throughout the United States. This crisis affects people with disabilities acutely, particularly those with intersecting forms of marginalization associated with low socioeconomic status, race, gender, and immigration status. Tackling the US housing crisis requires significant investment in affordable housing, including focused investments in accessible housing for people with disabilities. It also necessitates policy changes that address the ways in which current law, as well as its implementation and enforcement, prioritizes people or entities with power and privilege (e.g., property owners, housing developers, real estate investors, and landlords) to the detriment of people experiencing intersectional structural disadvantages (e.g., people with disabilities and people living in poverty). Although acknowledging that many major structural reforms are needed to fully realize access to safe, stable, accessible, and affordable housing for all people, especially those with disabilities, this article focuses on how equitable enforcement of existing laws can help to shift power to and protect the housing rights of people with disabilities.

Equitable enforcement strategies consider and seek to remedy the inequitable effects of enforcement practices on marginalized groups. For example, consistently enforcing housing safety laws in privileged communities while these laws go unenforced in low‐income communities of color has profound effects on health inequities.^3^ At the same time, enforcing criminal laws—such as prosecution for minor drug offenses—more aggressively in low‐income communities of color as compared with more affluent communities also has a detrimental effect on health in those communities.^3^ Because people with disabilities are disproportionately low‐income people of color, underenforcement of their housing rights and overenforcement of punitive laws in their communities disproportionately affects their health, safety, stability, and ability to thrive. Although *equal* enforcement of the law in all communities would improve health equity, *equitable* enforcement both prioritizes enforcement of protective housing laws in underserved communities and redirects enforcement resources from punitive policies that disproportionately harm these same communities to policies that reduce health inequities.

Framed through the lenses of disability and health justice, this article explores how two dimensions of inequitable enforcement—underenforcement of some laws and overenforcement of others—manifest, interact, and amplify one another in the context of housing policy, creating a vicious cycle of poverty, poor health, and marginalization for disabled populations. We propose solutions that policymakers should consider to facilitate equitable enforcement, which is the process of ensuring compliance with the law while considering and minimizing harms to marginalized people.^2^ We describe equitable enforcement strategies that both allocate resources toward enforcement of protective housing laws (e.g., antidiscrimination laws and housing safety codes) and minimize enforcement actions that exacerbate cycles of housing instability and homelessness for people with disabilities (e.g., eviction, chronic nuisance, and criminalization of homelessness). In the context of housing policy, equitable enforcement solutions for people with disabilities include proactive, preventive, and strategic approaches that protect safe, stable, and accessible housing and promote both disability and health justice.

## Disability and Health Justice Frameworks

### Disability Justice

There is no single definition of “disability.” Medical definitions differ from legal definitions. Federal laws apply different legal definitions and sometimes rely on the medical community to provide evidence that an individual meets the legal definition.^4^ In contrast, disability justice advocates reject the medicalization of disability and seek to enable people with disabilities to articulate their own experiences and the ways in which their experiences of disability are affected by social norms. In other words, disability justice rejects a medical model that “seeks to fix disability to the extent possible through a biomedical cure or remedy,”^5^ instead promoting a social model that “scrutinize[s] not bodily or mental impairments but the social norms that define particular attributes as impairments, as well as the social conditions that concentrate stigmatized attributes in particular populations.”^6^ Disability justice calls attention to the ways in which ableism, which defines and structures disability as outside the “norm,” excludes, devalues, and stigmatizes people with disabilities.^7^ It applies a cross‐disability and intersectional framework to combat exclusion of and discrimination against people with disabilities by centering their voices and lived experiences across race, socioeconomic status, gender, sexuality, and type of disability. Thus, disability justice exposes and seeks to dismantle intersectional systems of oppression by promoting self‐determination for people with disabilities while also recognizing the values of interdependence and collective access.^8^

### Health Justice

Like disability justice, the term, “health justice” has been used in recent years to structure discourse about who has the “opportunity to reach their full health potential” by recognizing that “individual and population health and well‐being are primarily driven by upstream structural factors … not by the individual behaviors and choices of those in poor health.”^5^ A health justice framework considers how laws, policies, and systems of power exclude and stigmatize people based on status—such as disability, race, gender, sexuality, and immigration status. It also considers how laws and policies function as both upstream drivers of health inequities and as necessary tools to reverse those drivers and reform systems so that everyone has an opportunity to be as healthy as possible. “Health justice addresses health inequities and health care inequalities by recognizing the human rights, civil rights, value and dignity of all people” and by promoting “self‐determination for those who have experienced exclusion, discrimination and stigma.”^5^ As further described below, inequitable enforcement of housing‐related laws presents one of the many challenges that people with disabilities experience that negatively affect their health and self‐determination.

## Background: Housing Safety, Stability, and Accessibility for People With Disabilities

Various intersectional factors exacerbate exclusion of and health inequities for people with disabilities, including poverty, racism, gender‐based discrimination, and ableism.^9^ People with disabilities are twice as likely as people without disabilities to live in poverty and three times as likely to experience food insecurity. The economic disparities are even greater for people of color with disabilities and people with mental health disabilities.^10^, ^11^

Nationally, there is a severe shortage of safe, affordable, and accessible housing—a problem that affects low‐income people with disabilities in particular and one that will likely worsen given an aging population.^12^, ^13^ A 2022 analysis found that there are only 36 affordable rental units available for every 100 extremely low‐income renter households and that 46% of extremely low‐income renter households include seniors or people with disabilities.^12^ People with disabilities also experience the highest rates of discrimination in housing transactions when compared with other protected groups. Fifty‐six percent of all housing discrimination complaints filed in 2018 alleged disability discrimination.^14^ A separate analysis found that “the vast majority of US homes lack basic accessibility features” and that approximately 3.5 million households headed by adults aged 65 years old and older reported having difficulties navigating or using their homes as of 2019.^13^

These intersectional factors, combined with the extreme housing shortage, lead to less housing opportunity, higher rates of housing insecurity, and higher rates of homelessness among people with disabilities. Federal Supplemental Security Income (SSI) benefits are insufficient to cover average rental costs for studios and one‐bedroom apartments, meaning many people with disabilities who lack additional income are priced out of safe and healthy housing.^15^ This can force low‐income people with disabilities into substandard housing or onto the streets. In 2017, the US Department of Housing and Urban Development (HUD) estimated that nearly 24% of people experiencing homelessness were people with disabilities.^16^ Losing housing makes it more difficult to find and keep a job, attend school, and access medical care and treatment—important factors that promote health and well‐being.^17^ Although the affordable housing crisis affects people across the United States, people with disabilities experience a disproportionate burden of inaccessible and unsafe housing and displacement leading to homelessness.

As noted earlier, addressing the affordable housing crisis and the particular injustices related to housing experienced by people burdened by intersectional disadvantage is beyond the scope of this article. Here, we consider the particular role of inequitable enforcement of existing laws and the need to repeal certain laws that, when enforced against people with disabilities, have an inequitable effect on their housing rights and health. Below, we describe the many barriers that people with disabilities experience in accessing safe and stable housing. Specifically, we probe the role of underenforcement of protective civil rights and housing laws and the overenforcement of punitive nuisance and criminal laws in further excluding and stigmatizing people with disabilities and exacerbating health inequities between disabled and nondisabled people. Inequitable enforcement exemplifies how systemic failures implicate health and disability justice.

## Underenforcement of Laws Protecting Housing Safety, Stability, and Access for People With Disabilities

Laws designed to facilitate the provision of safe and accessible housing, such as housing safety codes and civil rights laws prohibiting various forms of housing discrimination, are chronically underenforced. Underenforcement blunts the intended benefits of these laws, harming the health and well‐being of people with disabilities and further excluding them from full participation in society.^17^

### Access and Stability: Antidiscrimination Laws

**Figure 1:**
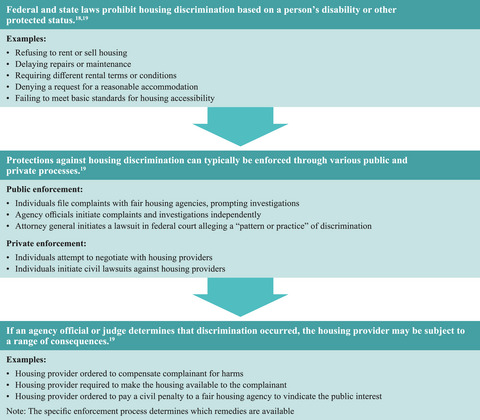
Enforcement of Laws Prohibiting Housing Discrimination [Colour figure can be viewed at wileyonlinelibrary.com]

A range of federal laws (e.g., the Fair Housing Act [FHA] and Section 504 of the Rehabilitation Act of 1973) and their state‐level counterparts promise equal opportunity for people with disabilities by prohibiting various kinds of housing discrimination (Figure 1).^18^, ^19^ Unfortunately, enforcement of these laws often falls short, as evidenced by widespread noncompliance.

For example, a 2017 HUD study focused on discrimination against people with mental health disabilities—specifically mental illness (MI) and intellectual and developmental disabilities (I/DD)—and found “significant levels of adverse differential treatment toward individuals with MI and I/DD when compared with individuals who did not have [mental disability].”^20^ Using paired testing (in which a person with disabilities and a person without disabilities inquire about the same housing unit), the study found that people with MI and I/DD were less likely to receive a response to their inquiry about the housing unit, less likely to be invited to inspect the unit, and more likely to be treated adversely both at the time of inquiry and when requesting accommodation.^20^ A 2015 testing study led by the HUD and the Urban Institute similarly found that deaf and hard‐of‐hearing people experience barriers when seeking housing, including difficulties communicating with housing providers. They also found that people in wheelchairs experienced problems at “several points in the process, including finding accessible units, securing appointments with providers and being shown units, and receiving a definite response to their reasonable modification requests.”^21^ Other independent investigations have shown that certain cities frequently failed to ensure that developments receiving federal funding satisfied design and construction standards established by the FHA and Section 504 to ensure a basic level of accessibility, spurring advocates to pursue legal challenges with some degree of success.^22^, ^23^ Unless more government resources are committed to enforcement, violations of fair housing laws and accessibility standards are unlikely to be identified and enforced consistently, depriving people with disabilities of access to safe, stable, and accessible housing.

One form of prohibited discrimination under the FHA and Section 504 is for a housing provider to deny a request for a reasonable accommodation in rules, policies, practices, or services when such an accommodation is necessary to afford a person with a disability equal opportunity to use and enjoy a dwelling.^18^, ^19^ The obligation to make reasonable accommodations can fulfill needs that are not covered by design and construction standards, such as allowing an assistance animal in a “no‐pets” building or aligning rent payment schedules with the allocation of income assistance.^19^ A housing provider generally may not refuse a request for a reasonable accommodation unless it would create an “undue” financial burden or fundamentally alter the provider's services.^24^ The actual or perceived cost of granting reasonable requests—as well as widespread suspicion of “fakery” regarding people with disabilities’ needs^25^—may influence how landlords act on their legal obligations. Of all complaints of disability‐based housing discrimination filed between 2008 and 2018, allegations that housing providers failed to make reasonable accommodations were the most common concerns.^26^

The right to reasonable accommodations in housing is underenforced for a variety of reasons, from limited public education and weak notice requirements that could increase knowledge of antidiscrimination protections to a lack of affordable civil legal assistance to help people request accommodations or file complaints that challenge wrongful denials. Ten percent of all housing discrimination complaints received by fair housing agencies in 2020, including those based on disability, were closed for administrative reasons that competent legal representation could help to avoid.^27^ Insufficient investment in public enforcement also plays a role. In 2018, private nonprofit fair housing organizations addressed three times as many housing discrimination complaints as all government agencies combined at a time when those agencies were experiencing funding and regulatory challenges.^14^ Agencies and organizations that investigate complaints are often responsible for civil rights enforcement generally and “may miss the complexities and nuances necessary to understand the need for reasonable accommodations” because of a lack of specialized training in disability issues.^26^

In short, despite existing fair housing laws, a significant number of people with disabilities live in housing that poorly suits their needs. Insufficient public investment, barriers to exercising fair housing rights, and resulting underenforcement are among the many structural factors that contribute to this problem.

### Health and Safety: Housing Code Provisions

**Figure 2:**
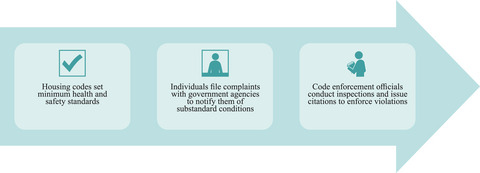
Complaint‐Based Enforcement of Housing Health and Safety Codes [Colour figure can be viewed at wileyonlinelibrary.com]

Housing codes establish standards for health and safety that landlords must meet in order to offer a property for rental. Although housing codes are commonplace throughout the United States, substandard housing continues to be a problem for more than 3.3 million families.^28^ People with disabilities are more likely to live in unsafe housing that adversely effects their health than people without disabilities.^29^ Thus, underenforcement of housing codes raises both disability and health justice concerns. Most jurisdictions rely on individual complaints to identify and enforce housing code violations (Figure 2), which can leave people with disabilities and other vulnerable populations behind.^30^ As with the right to request reasonable accommodations, people with disabilities face structural barriers to actualizing the guarantee of safe housing via the complaint process, including a lack of knowledge about when and how to file complaints, communication barriers, fear of landlord retaliation, and lack of access to affordable civil legal assistance. Although tenants may seek to enforce housing standards on their own through negotiation or affirmative litigation,^28^ the same structural barriers adhere. As a result, the legal guarantee of safe and healthy rental housing often goes unenforced and unfulfilled for people experiencing intersectional structural disadvantages, including disability.^30^

When the housing rights of people with disabilities go unenforced or underenforced, the structural factors discussed above—from poverty to housing supply shortages—leave them with few options. An individual may have to choose between remaining in inadequate or unhealthy housing or leaving with no guarantee of finding a suitable alternative. If people with disabilities remain in substandard or inaccessible housing, the consequences can be especially severe, such as living in conditions that cause or exacerbate health problems (e.g., mold or pests exacerbating asthma and respiratory distress),^31^ experiencing serious safety risks (e.g., being trapped inside a building or unable to access a unit because an elevator has not been repaired),^32^ or being unable to live independently or manage everyday tasks (e.g., inability to bathe because a shower lacks grab bars).^33^ Consistent and equitable enforcement of housing codes, accessibility standards, reasonable accommodations, and other housing laws is essential to prevent these outcomes and ensure that the intended benefits of protective housing laws are realized.

## Misapplication and Overenforcement of Laws That Harm Housing Safety, Stability, and Access for People With Disabilities

Even as people with disabilities are frequently excluded from the benefits of protective housing laws, they are also disproportionately subject to punitive laws related to housing. The overenforcement of these laws can result in housing loss and increased instability in individuals’ lives, compounding existing health problems. People with disabilities commonly face overenforcement of two interrelated areas of the law: chronic nuisance ordinances (CNOs) and eviction.

### CNOs

An increasing number of local jurisdictions are passing CNOs, which are intended to protect tenants from frequent disruption to the use and enjoyment of their homes. The ordinances typically require landlords to take action to abate nuisance activities, up to and including evicting tenants. Although “nuisance” can be broadly defined in these ordinances as any activity interfering with the enjoyment of one's home, some jurisdictions specifically declare actions like frequent 911 calls, excessive noise, or frequent destruction of property to be chronic nuisances.^34^

**Figure 3:**
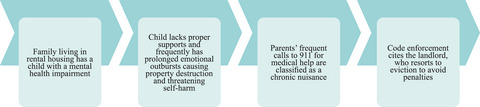
Enforcement of Chronic Nuisance Ordinance Against Persons With Disabilities and Their Families [Colour figure can be viewed at wileyonlinelibrary.com]

Defining nuisance in this way can unfairly include behaviors related to disabilities. Because such ordinances rarely have exceptions for people with mental health disabilities, medical emergencies, or chronic medical conditions, the ordinances are often inequitably overenforced against individuals with mental health impairments and their families (as well as people of color and survivors of domestic violence), putting them at increased risk of housing loss and instability (Figure 3).^34^ Furthermore, rather than utilizing government resources upstream to address the underlying health needs of people with disabilities and allow families to maintain stable housing, CNOs shift responsibility to property owners, obliging them to take an active role in responding to so‐called nuisance activities.

### Eviction

Each year in the United States, 7.6 million people are threatened with eviction from their homes. More than half (3.9 million people) are part of a household that experiences an eviction judgment.^35^ People with disabilities may be more susceptible to eviction than other tenants. For example, a person in a wheelchair who does not have adequate support in maintaining their home may be evicted for violating maintenance‐related lease provisions. People with disabilities who depend on SSI benefits may have difficulty paying rent after an increase, leaving them vulnerable to eviction for nonpayment. Indeed, people with disabilities report high levels of housing insecurity.^36^ Disabled renters of color are disproportionately affected.^36^

A person's disability may also impair their ability to effectively counter eviction threats, making housing loss more likely. An eviction notice or unlawful detainer often requires a quick response from the tenant to contest the eviction or establish affirmative defenses. A timely response may be impossible, however, if a tenant has difficulty understanding the notice or filing court paperwork because of a cognitive impairment, limited mobility, or hospitalization because of their disability. Power imbalance between low‐income tenants and property owners, who are much more likely to be represented by counsel, leaves many disabled tenants without protection. Ninety percent of landlords are represented in eviction proceedings, whereas only 10% of tenants are.^37^

During legal proceedings related to an eviction, a person with a mental health impairment may exhibit behaviors because of their disability that, without appropriate advocacy on their behalf or adequate training for the judiciary,^10^ may bias a judge against them.^38^ Eviction, or even just the threat of eviction, is associated with myriad negative health impacts, including increased risk of hospitalization and disruptions to health care access.^17^, ^39^ For example, an individual who loses their housing could miss Medicaid recertification paperwork, or a child receiving in‐home behavioral therapy may be unable to maintain their treatment plan. In addition, “the health conditions and high health care costs associated with eviction make future evictions more likely,” further exacerbating health problems and the cycle of poverty.^39^ Abelist approaches to underenforcement of protective laws and overenforcement of punitive laws reinforce disability and health injustice.

## A Vicious Cycle: The Compounding Effects of Under‐ and Overenforcement

**Figure 4:**
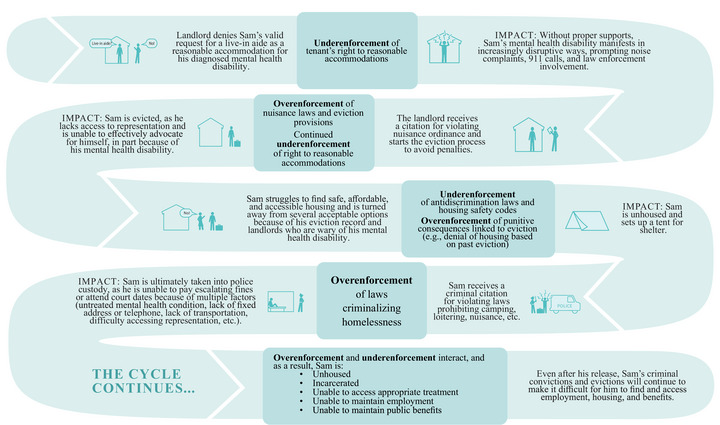
The Vicious Cycle of Underenforcement and Overenforcement of Housing‐Related Laws [Colour figure can be viewed at wileyonlinelibrary.com]

The combination of underenforcement of protective housing laws and the misapplication and overenforcement of punitive housing laws often interact, compounding exclusion, stigma, and poor health for people with disabilities (Figure 4). For example, the underenforcement of antidiscrimination and housing safety laws can leave people with disabilities susceptible to eviction or unable to access adequate housing, in turn making them more likely to experience homelessness. Once unhoused, people with disabilities may be subjected to misapplication and overenforcement of laws, adopted in many jurisdictions, that criminalize activities like loitering, panhandling, or camping, sitting, or lying down in public places.^40^ A study using 2016 data from the Bureau of Justice Statistics found that 40% of people in state and federal prisons reported that they had a psychiatric disability, whereas 57% reported a nonpsychiatric disability.^41^ Moreover, 42% of federal and state prison populations consisted of people of color with disabilities.^41^ Poverty and homelessness, which people with disabilities disproportionately experience, can increase the likelihood of encounters with law enforcement and incarceration, especially for people of color.^10^, ^11^, ^16^, ^42^

Overenforcement of criminal laws against people with disabilities also means that they will be burdened by a criminal record, which, like a record of eviction, can make finding housing extremely challenging, if not impossible, in the future. This process creates a vicious cycle of housing instability as described in Figure 4.

## Enforcement Problems and Solutions

Inequitable enforcement of housing‐related laws negatively affecting people with disabilities is a systemic problem that requires systemic solutions.

### Addressing Underenforcement: Removing Barriers to Exercising Existing Rights

Traditionally, governments use individual complaints to identify and address housing discrimination and safety issues. This approach puts the onus on people with disabilities to assert their rights, often without adequate support and resources to navigate complaint processes or recognize unlawful conduct in the first place. Relatedly, when faced with eviction, people with disabilities often experience challenges exercising their rights because notice is inaccessible (e.g., for people with limited English proficiency, low vision, or cognitive challenges) and the required timeframe to respond is brief—often just 3 days. Below, we identify promising policies that shift the burden of ensuring legal compliance from individuals to government entities and that empower people with disabilities to exercise their legal rights directly.

*Proactive and Strategic Approaches to Housing Discrimination*. The pilot testing studies discussed earlier demonstrate that disability‐based housing discrimination is a significant problem. Local governments and fair housing organizations can implement testing programs as one strategy to proactively combat such discrimination. Regular, systematic testing can help to identify instances and patterns of discrimination even without individual complaints.^43^ Further, such programs can aid in prioritizing enforcement against repeat offenders. Testing for discrimination can be coupled with public education for housing providers to prevent recurring violations and promote compliance with fair housing laws before violations occur. Systematic testing is also invaluable in identifying discrimination in lending, in which complex practices pose a barrier to filing complaints.^43^

State Attorneys General (AGs) play a critical role in the strategic enforcement of state‐level antidiscrimination laws. For example, in 2021, Virginia's AG partnered with nonprofit groups to file the state's first lawsuits alleging that housing providers had engaged in a “pattern or practice” of discrimination based on race, gender, and source of income (which may overlap with disability with respect to disability‐based benefits) in violation of the Virginia Fair Housing Law.^44^ California's AG recently created a “Housing Strike Force” and plans to convene tenant roundtables across the state. These efforts seek to inform tenants and homeowners of their housing rights and identify opportunities for the AG's office to enforce housing protections for California residents.^45^ Proactive and strategic use of state resources can achieve a larger benefit than individual complaints and lawsuits. Combating discrimination not only ensures that appropriate housing is available but also helps to preserve the self‐determination and dignity of people with disabilities.

*Proactive and Strategic Approaches to Housing Code Enforcement*. In the context of housing code enforcement, proactive rental inspection (PRI) programs ensure regular, periodic inspections of all covered properties regardless of whether individual tenants have complained. PRI policies can effectively reduce health and safety risks associated with housing code violations and can help protect people who are often underserved by governments and institutions.^30^ PRI programs work best when coupled with a complaint‐based system, as individual complaints often identify pressing issues in a more timely manner and prompt enforcing agencies to investigate issues that may affect multiple households, such as mold or lead paint.

Additionally, localities should consider best practices that promote compliance by property owners and improve outcomes while avoiding some of the unintended negative consequences of punitive code enforcement, such as increased rents or removal of properties from the rental market. Best practices can include, for example, implementing PRI in conjunction with programs to support displaced tenants and assist landlords with repairs (e.g., Los Angeles’ Rent Escrow Account Program) or adopting performance‐based strategies that lower fees and reduce the frequency of inspections for compliant landlords while simultaneously requiring larger fees and more frequent inspections for repeat violators.^30^ PRI requires a shift in priorities and practices for housing code enforcement agencies. It also necessitates increased funding to support additional staff. But investment in proactive approaches can reduce downstream costs associated with housing‐related health harms and homelessness.

Housing code enforcement agencies can also use data to improve allocation of resources and services to meet the needs of people with disabilities. For example, such agencies can prioritize proactive inspections in geographic areas with disproportionately high numbers of tenants with disabilities and lower income levels, ensuring that limited resources have the most impact.^30^, ^46^ Interagency data sharing is another promising tool. In the City of Elmira, New York, the Department of Social Services (DSS) partnered with code enforcement to maintain a list of problem landlords. The DSS uses this list to ensure that residents who receive emergency assistance from public housing authorities are not placed with landlords with a history of code violations. Code enforcement also provides notice to the DSS before condemning properties used as boarding houses to protect tenants from forced relocation. This cross‐agency collaboration prevents property owners from taking advantage of residents with housing vouchers and ensures the DSS placements are safe and stable.^47^

It is also critically important to increase resources for training code enforcement officers in legal and technical issues, customer service, and cultural sensitivity. Code enforcement officers who understand the housing needs of people with disabilities—and the health and safety risks of failing to meet those needs—are better equipped to enforce housing codes and achieve the intended policy goals.^47^ Enforcing agencies can also build capacity through partnerships with community‐based organizations that serve and have already built trust with people with disabilities and other residents.^30^

*Investment in Access to Legal Assistance*. As described above, eviction can lead to a vicious cycle of unstable housing, unaddressed health needs, homelessness, and incarceration for people with disabilities. Research shows that legal representation significantly improves outcomes for low‐income tenants facing eviction, increasing their likelihood of staying in their homes.^48^ In line with this research, a number of state and local jurisdictions across the country have adopted laws providing tenants with a right to counsel in eviction hearings,^49^ and preliminary data on some of those programs show that the laws have successfully reduced eviction rates.^50^ Legal aid programs, which offer legal assistance to low‐income people without charge, also provide services that can decrease the risk of eviction occurring at all. Such services include helping tenants file complaints regarding housing discrimination and safety violations and advocating to stabilize disability‐based benefits and housing subsidies.

Despite the benefits of representation, access to legal assistance remains limited in part because legal aid programs have been chronically underfunded by the federal agency that provides most of their resources.^51^ Medical–legal partnerships (MLPs), which integrate civil legal services into health care settings for low‐income and marginalized populations, are one way to increase access to justice for people with disabilities. By identifying unmet social and legal needs at medical visits, MLPs can prevent housing problems before they become acute, thereby helping to prevent eviction, remedy health‐harming illegal housing conditions, and support disabled tenants to request accommodations. Because MLP advocates partner with clinicians, they have direct access to supportive medical evidence when making requests for reasonable accommodations; this improves the likelihood of obtaining accommodations and directly addresses issues related to housing safety and discrimination.^51^ MLPs also help to educate patients about their legal rights under housing and disability laws, empowering them to advocate for themselves. Policymakers can support the development of MLPs in their states and localities. Recently, Congress appropriated funding in the fiscal year 2023 Omnibus Appropriations bill for an MLP grant program through the US Department of Health and Human Services.^52^

### Addressing Overenforcement: Rejecting Punitive, Health‐Harming, and Stigmatizing Approaches

Rather than enforcing protective laws and using government resources to support housing security and safety, governments often employ scarce resources to enforce punitive laws (e.g., nuisance, eviction) that can lead to housing instability and homelessness for people with disabilities. The following strategies shift away from punitive enforcement in favor of more proactive and preventive measures.

*Repeal or Preempt CNOs*. To reduce eviction rates, some states have enacted legislation requiring municipalities to amend their nuisance ordinances to exempt 911 calls related to domestic violence survivors and people with disabilities.^34^ However, even when such legislation is passed, research has found ongoing CNO enforcement against people with disabilities in violation of the exceptions.^34^ More effective interventions include amending CNOs to omit eviction as an enforcement option, repealing CNOs entirely, or preempting local CNOs at the state level.

At the federal level, the most recent reauthorization of the Violence Against Women Act^53^ protects an individual's right to report crime from their own home. If the resident is not at fault, a government entity that receives housing and community development funds may not impose fines or fees on them, evict them, or refuse to rent to them or to renew their tenancy for seeking emergency assistance or law enforcement in response to criminal activity. These protections have particular relevance for people with disabilities, who experience violence at disproportionate rates.^54^ To further support people with disabilities, state and local jurisdictions can adopt laws that extend similar protections to all types of rental housing and that expand the scope of the protections to include people seeking emergency assistance or law enforcement for reasons unrelated to criminal activity, such as medical emergencies.

*Decriminalizing Disability by Decriminalizing Homelessness*. As discussed earlier, people with disabilities—particularly people with mental health disabilities—are overrepresented among people experiencing homelessness and dramatically so among people who are incarcerated.^16^, ^41^ Thus, criminalizing homelessness often means criminalizing disability. Criminal sanctions do nothing to address the root causes of homelessness and can jeopardize access to government benefits, interrupt treatment, impede housing access and stability, and exacerbate physical illness and MI. Policymakers can enact laws ensuring that people with pertinent training respond to incidents involving homeless people with mental health problems. Furthermore, they can invest in diversion programs that direct people to appropriate supportive services rather than incarceration. Below, we highlight successful examples of such approaches.

Embedding mental health and disability advocates in police departments—or establishing partnerships between law enforcement and other agencies with relevant expertise—can improve emergency responses to mental health, homelessness, or substance use–related incidents. The City of Denver's Support Team Assisted Response (STAR) is an exemplar in which emergency medical technicians and behavioral health clinicians partner to respond to 911 calls. Between 2020 and 2022, STAR teams responded to over 2,200 calls and never called for police backup because of safety concerns. The program is expected to expand in Denver and other cities across the state based on its demonstrated success.^55^ In addition to reducing the harms of criminal enforcement for people with disabilities and unhoused individuals, the program also frees capacity for law enforcement to respond to high‐priority calls. This type of partnership can also be responsive to intersectional implications of overpolicing in communities of color.

Diversion programs that address mental health, substance use, and disability‐related needs are another effective tool to avoid criminal sanctions. Areas of focus include wraparound case management, vocational rehabilitation, and referrals to supportive services and treatment. For example, Buncombe County, North Carolina, recently launched the Unhoused Diversion Program, an initiative designed to address the needs of unhoused people charged with minor, nonviolent crimes.^56^ The goal is to provide resources that keep people out of jail and support their ability to thrive as supported and integrated members of the community. Given the significant overlap between the criminalization of homelessness and disability, these types of diversion programs could play an important role in stabilizing housing, reducing poverty, and preventing health harms for people with disabilities.

## Conclusion

Ultimately, the inequitable enforcement of housing‐related laws as applied to people with disabilities reflects policy choices. By investing in punitive and criminal enforcement of housing‐related laws while insufficiently investing in the enforcement of laws that promote housing safety, stability, and accessibility, governments create a vicious cycle that harms health and opportunity for residents who are most in need of public support and resources.

Reversing these policy priorities and implementing the equitable strategies identified above will require greater government investment. Other potential barriers include political feasibility, stigma related to disability and poverty, insufficient capacity at enforcing agencies, and a still‐nascent evidence base for equitable enforcement strategies. Jurisdictions willing to pilot and evaluate enforcement strategies that prioritize health and disability justice will be critically important to increase their adoption.

In the long term, stable housing that properly accommodates people with disabilities will improve health, increase employment, decrease the need for emergency services, and reduce incarceration, promoting health justice for people with disabilities and their communities.

## Funding/Support

Support for this research was provided by the Robert Wood Johnson Foundation (award 79166). Note that the views expressed in this article do not necessarily reflect the views of the Robert Wood Johnson Foundation.

## Conflict of Interest Disclosures

No disclosures were reported.
